# Isoalantolactone Inhibits UM-SCC-10A Cell Growth via Cell Cycle Arrest and Apoptosis Induction

**DOI:** 10.1371/journal.pone.0076000

**Published:** 2013-09-30

**Authors:** Minjun Wu, Hua Zhang, Jiehua Hu, Zhiyong Weng, Chenyuan Li, Hong Li, Yan Zhao, Xifan Mei, Fu Ren, Lihua Li

**Affiliations:** 1 Department of Cell Biology and Anatomy, Liaoning Medical University, Jinzhou, China; 2 Educational Technologies and Simulation Training Centre, Naval University of Engineering Tianjin Campus, Tianjin, China; Rajiv Gandhi Centre for Biotechnology, India

## Abstract

Isoalantolactone is a sesquiterpene lactone compound isolated from the roots of *Inula helenium* L. Previous studies have demonstrated that isoalantolactone possesses antifungal, anti-bacterial, anti-helminthic and anti-proliferative properties in a variety of cells, but there are no studies concerning its effects on head and neck squamous cell carcinoma (HNSCC). In the present study, an MTT assay demonstrated that isoalantolactone has anti-proliferative activity against the HNSCC cell line (UM-SCC-10A). Immunostaining identified that this compound induced UM-SCC-10A cell apoptosis but not necrosis. To explain the molecular mechanisms underlying its effects, flow cytometry and western blot analysis showed that the apoptosis was associated with cell cycle arrest during the G1 phase, up-regulation of p53 and p21, and down-regulation of cyclin D. Furthermore, our results revealed that induction of apoptosis through a mitochondrial pathway led to up-regulation of pro-apoptotic protein expression (Bax), down-regulation of anti-apoptotic protein expression (Bcl-2), mitochondrial release of cytochrome c (Cyto c), reduction of mitochondrial membrane potential (MMP) and activation of caspase-3 (Casp-3). Involvement of the caspase apoptosis pathway was confirmed using caspase inhibitor Z-VAD-FMK pretreatment. Together, our findings suggest that isoalantolactone induced caspase-dependent apoptosis via a mitochondrial pathway and was associated with cell cycle arrest in the G1 phase in UM-SCC-10A cells. Therefore, isoalantolactone may become a potential drug for treating HNSCC.

## Introduction

The sixth most common form of cancer worldwide is head and neck cancer [1], of which 90% of cases are head and neck squamous cell carcinoma (HNSCCs), which refers to squamous cell carcinoma (SCC) arising from the mucosal surfaces of the oral cavity, oropharynx, larynx or hypopharynx. HNSCC is the eighth most common cause of mortality due to cancer worldwide and is known to be difficult to treat; consequently, it has only a 50% five-year survival rate [2]. During the past few decades, aggressive and combined treatment regimens have been used, including chemoradiation, radical surgery, and neoadjuvant chemotherapy, depending on the site, size and stage of the lesions. Despite the considerable advances in diagnostic and therapeutic measures, the prognosis of HNSCC remains poor. Surgery is usually performed for early-stage disease, and radiotherapy always has a variety of severe adverse affects. Therefore, developing novel chemotherapeutic agents with less toxicity and understanding their molecular mechanisms are necessary for improving HNSCC outcomes.

Plants are considered to be one of the most important sources of anticancer drugs. We performed high throughput screening of a compound library of Chinese herbs to identify anti-HNSCC compounds. Isoalantolactone, a sesquiterpene lactone, showed anti-tumor effects against an HNSCC cell line (UM-SCC-10A). Currently, several sesquiterpene lactone compounds, which are plant compounds, are seen as one of the most important sources of potential anticancer agents, and have been used in cancer clinical trials for breast, colorectal, kidney, prostate, acute myeloid leukemia, acute lymphoblastic leukemia, non small lung cancer [3], [4], gynecologic tumors [5] and pancreatic cancer [6]. In addition, other studies have reported that isoalantolactone, isolated from the roots of *Inula helenium* L., possesses antifungal, anti-bacterial, anti-helminthic and anti-proliferative activities [7]. However, the effects of isoalantolactone and its mechanism of action on human head and neck squamous cell carcinoma have not been studied.

In present studies, we investigated the potential anti-tumor effects of isoalantolactone on UM-SCC-10A cells. An MTT assay, a Live/Dead cell assay, Hoechst33258 staining, cell cycle and apoptosis assays and analysis of apoptosis regulator expression were performed. We found that isoalantolactone inhibited UM-SCC-10A cell growth. The common modes of cell death were necrosis, apoptosis and autophagy [8], [9]. We then identified isoalantolactone-induced UM-SCC-10A cell death by measuring cell apoptosis and cell cycle arrest in the G1 phase. Furthermore, the molecular mechanism for apoptosis was analyzed by determining the expression of apoptosis regulators using western blotting. The results indicate that isoalantolactone induced caspase-dependent apoptosis via a mitochondrial pathway and was associated with cell cycle arrest in the G1 phase in UM-SCC-10A cells. Our studies will help identify and screen key target molecules to treat HNSCC.

## Materials and Methods

### Materials

Isoalantolactone was obtained from the National Institute for the Control of Pharmaceutical and Biological Products in China (purity >99% as determined by analytical HPLC). Propidium iodide (PI), Hoechst33258, dimethylsulfoxide (DMSO), [3-(4,5-dimethylthiazol-2-yl)-2,5-diphenyltetrazolium bromide] (MTT), Z-VAD-FMK, Dulbecco's Modified Eagle's Medium (DMEM), fetal bovine serum (FBS), RNase A, penicillin and streptomycin were purchased from Sigma Chemical Co. (St. Louis, MO, USA). Rhodamine 123 was purchased from Eugene Co. (OR, USA). The annexin V-FITC apoptosis detection kit was purchased from Beyotime Institute of Biotechnology (Shanghai, China). Mouse polyclonal anti-human Bcl-2, rabbit polyclonal anti-human Bax, cytochrome c, p53, p21, cyclin D and caspase-3 antibodies were purchased from Cell Signaling Technology (Beverly, MA, USA). Antibodies specific to β-actin and horseradish peroxidase-conjugated secondary antibodies (goat-anti-rabbit, goat-anti-mouse) were purchased from Santa Cruz Biotechnology (Santa Cruz, CA, USA).

### Cell Culture and Treatment

The UM-SCC-10A cell line was purchased from the Shanghai Institute of Biological Science (Shanghai, China). The cells were grown in plastic culture flasks under standard conditions (37°C with 5% CO_2_ in a completely humidified atmosphere) using DMEM medium supplemented with 10% heat-inactivated FBS, 2 mM L-glutamine, 100 U/ml penicillin and 100 g/ml streptomycin. Cell detachment was achieved by rinsing with 0.05% trypsin/0.02% EDTA solution. After 24 h of attachment, the cells were treated with isoalantolactone at an IC_50_ concentration for 24 h, except for the cell proliferation assay.

### Splenocytes Isolation

All animal procedures were approved by the Experimental Animal Committee of Liaoning Medical University. 8 week old C57/BL6 mouse was used in this experiment. Mouse was anesthetized using Pentobarbital sodium (65 mg/kg ip) and perfused transcardially with PBS. Following midline abdominal incision spleen was removed and isolated freshly splenocytes and cultured in RPMI 1640 medium supplemented with 20% heat-inactivated FBS and maintained at 37°C with 5% CO_2_ in a completely humidified atmosphere.

### Cell Proliferation Assay

The cell proliferative effect of isoalantolactone was evaluated using an MTT assay in UM-SCC-10A cell line. The cells were seeded onto 96-well plates at 5×10^3^ cells/well and incubated at 37°C for 24 h. DMSO or different concentrations of isoalantolactone (0 to 100 µM) were subsequently added and incubated for another 24 to 48 h. The cells were further incubated with 10 µl MTT solution (5 mg/ml) at 37°C for 4 h. After removing the medium, 150 µl DMSO was added in each well, and the plates were shaken for 15 min to dissolve the formazen crystals. The absorbance wavelength at 570 nm was measured for each well in a thermo-microplate reader (Thermo Scientific, USA). The results were calculated as follows:

Cell viability (%) = (A570 sample – A570 blank)/(A570 control – A570 blank)×100.

### Live/Dead Cell Assay

The living and dead cells were quantified using trypan blue staining and a cell viability analyzer (Beckman Coulter, Epics XL, USA). To determine the effect of isoalantolactone, UM-SCC-10A cells were incubated with 25 or 50 µM isoalantolactone for 24 h. Subsequently, the cells were collected and washed with PBS and then incubated with 0.04% trypan blue for 5 min at room temperature. Trypan blue only stains dead cells. After washing, the cells were resuspended in PBS and the percentage of living and dead cells were counted. The living cell rate (%) = number of living cells/(number of living cells+number of dead cells)×100.

### Apoptosis Analysis

The apoptotic cells were quantified using the annexin V and PI double staining kit. Briefly, UM-SCC-10A cells were treated with 25 or 50 µM isoalantolactone for 24 h. After treatment, the cells were collected, washed with PBS, and resuspended in 200 µl binding buffer containing 5 µl annexin V (10 µg/ml) for 10 min in the dark. The cells were then incubated with 10 µl PI (20 µg/ml), and the samples were immediately analyzed using flow cytometry (Beckman Coulter, Epics XL, USA). For the caspase inhibitor analysis, the cells were pretreated with 50 µM Z-VAD-FMK for 2 h and then incubated with 50 µM isoalantolactone. Data acquisition and analysis were performed using CellQuest software [10].

### DNA Fragmentation Assay

UM-SCC-10A cells were treated with 0, 25 or 50 µM isoalantolactone for 24 h. DNA fragmentation was measured using Hoechst33258 staining. The cells were fixed with 4% paraformaldehyde for 15 min at room temperature. Then, washing with PBS, the cells were stained with Hoechst33258 (50 µg/ml) at 37°C in the dark for 20 min. The cells were washed and resuspended in PBS to assess nuclear morphology under fluorescence microscopy.

### Cell Cycle Analysis

The DNA content of cells in the G0/G1, S and G2/M phases can be measured using flow cytometry. UM-SCC-10A cells were incubated with 25 or 50 µM isoalantolactone for 24 h. After treatment, the cells were collected, washed with PBS containing 2% FBS. 2×10^6^ cells/ml were fixed with cold absolute ethanol overnight at 4°C in a 15 ml polypropylene and V-bottomed tube. After washing with PBS twice, the cells were incubated with 1 ml PI staining solution (3.8 mM sodium citrate, 20 µg/ml PI in PBS). Add 50 µl of RNase A solution (10 µg/ml RNase A) and incubated for 30 min at room temperature in the dark. The DNA contents were analyzed by flow cytometry (Beckman Coulter, Epics XL, USA). For the flow cytometric analysis, at least 10,000 cells were used for each sample. The data were analyzed using Cell Quest software.

### Mitochondrial Membrane Potential Measurement

The mitochondrial membrane potential (MMP) was measured using rhodamine 123 (Rho123) retention in UM-SCC-10A cells. Rho123 is a membrane permeable fluorescent cationic dye. The Rho123 uptake by the mitochondria is proportional to the MMP. The cells were treated with 25 or 50 µM isoalantolactone for 24 h. After treatment, the cells were collected in centrifuge tubes and incubated with Rho123 (0.5 µM) for 20 min at room temperature in the dark. After being washed with PBS, the cells were resuspended in 800 µl PBS and analyzed using flow cytometry with excitation and emission wavelengths of 488 and 530 nm, respectively.

### Immunoblotting Analysis

UM-SCC-10A cells were treated with isoalantolactone for 24 h, washed twice with PBS, and lysed for 30 min on ice using WIP cell lysis reagent (20 mM Tris, pH7.5, 150 mM NaCl, 1% Triton X-100, 1 mM Na_2_EDTA, 1 mM EGTA, 2.5 mM sodium pyrophosphate, 1 mM β-glycerophosphate, 1 mM Na_3_VO_4_ and 1 µg/ml leupeptin). The insoluble protein lysate was removed by centrifugation at 12000 *rpm* for 15 min at 4°C. The protein concentrations were determined using a NanoDrop 1000 spectrophotometer (Thermo Scientific, USA). Forty µg proteins were electrophoresed using 10% SDS-PAGE and transferred to a PVDF membrane. After blocking with 5% (w/v) non-fat milk and washing with Tris-buffered saline-Tween solution (TBST), the membranes were incubated overnight at 4°C with specific primary antibodies and anti-rabbit IgG or anti-mouse IgG secondary antibodies for 1 h at room temperature. The signals were detected using an ECL plus chemiluminescence kit and X-ray film (Millipore Corporation, Billerica, USA). All of the bands were quantified by densitometry using Image J software.

### Statistical Analysis

All of the data are presented as the mean ± SEM and were analyzed using Students *t* test or by one-way ANOVA followed by Tukey’s multiple comparisons test. *P*<0.05 was considered to be statistically significant.

## Results

### Isoalantolactone Inhibits Cell Growth in a Tumor Selective Manner

**Figure 1:**
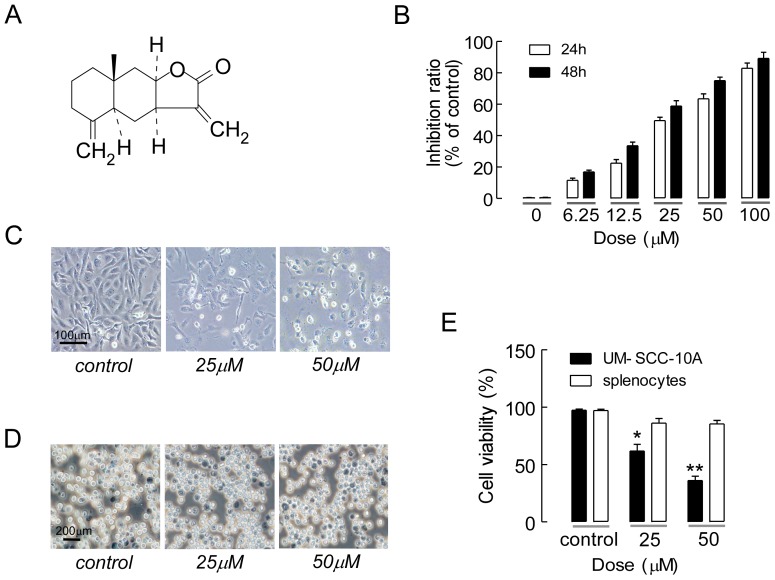
The chemical structure of isoalantolactone and its growth-inhibiting effect on UM-SCC-10A cells and mouse splenocytes. (A) The chemical structure of isoalantolactone. (B) UM-SCC-10A cells were pretreated with 0.1% DMSO or various concentrations of isoalantolactone for 24 and 48 h, and cell growth inhibition assays were performed using the MTT method. The data are expressed as the mean ± SEM of three independent experiments. (C) Isoalantolactone-induced morphologic changes in the UM-SCC-10A cells, and these changes were observed using inverted phase contrast microscopy. The control cells adhered well and displayed normal UM-SCC-10A cell morphology. After isoalantolactone treatment, many cytoplasmic vacuoles were observed in the cells. The vacuoles became progressively larger and denser with increasing concentrations of isoalantolactone. (D) Mouse splenocytes were treated with 25 and 50 µM isoalantolactone for 24 h and stained with 0.4% trypan blue, after which they were examined for dead and living cells microscopically. The dead cells stained blue. (E) Cell viability after 25 or 50 µM isoalantolactone treatment in UM-SCC-10A cells and mouse splenocytes for 24 h. The results are the mean ± SEM from three independent experiments. *P<0.05 and **P<0.01 compared to the control.

The UM-SCC-10A cell line and normal mouse splenocytes were used to assess the anti-proliferative effects of isoalantolactone, the chemical structure of which is shown in Figure 1A. The MTT assay showed that isoalantolactone inhibited cell growth in a dose- and time-dependent manner (Figure 1B). After the cells were treated with isoalantolactone for 24 and 48 h, the IC_50_ values were 50 µM and 25 µM, respectively. We observed cell morphology using inverted phase contrast microscopy. The cells adhered well and displayed normal morphology in the control group, whereas abundant cytoplasmic vacuoles were observed in the isoalantolactone-treated groups (Figure 1C). Moreover, the vacuolization in the cytoplasm became progressively larger and denser with increasing concentrations of isoalantolactone. After treatment with 50 µM isoalantolactone, the majority of cells became round and shrunken, and were observed to float in the culture medium, demonstrating that isoalantolactone inhibited tumor cell growth in a time-dependent manner. We also tested the cytotoxic effects of isoalantolactone in normal mouse splenocytes using trypan blue. Less inhibition was found in the splenocytes treated with isoalantolactone, suggesting that isoalantolactone has tumor cell selectivity (Figure 1D).

We further confirmed the effect of isoalantolactone-induced inhibition of UM-SCC-10A cell growth by analyzing the percentages of living and dead cells. Figure 1E shows the survival ratio of UM-SCC-10A cells treated with 25 or 50 µM isoalantolactone for 24 h. Compared to the control group, the number of living cells in the isoalantolactone-treated groups was significantly decreased in a dose-dependent manner. The number of living and dead cells was measured using a cell viability analyzer. Furthermore, isoalantolactone markedly inhibited UM-SCC-10A cell viability; thus, isoalantolactone may be a potent anti-tumor agent for HNSCC.

### Isoalantolactone Induces Cell Apoptosis

**Figure 2:**
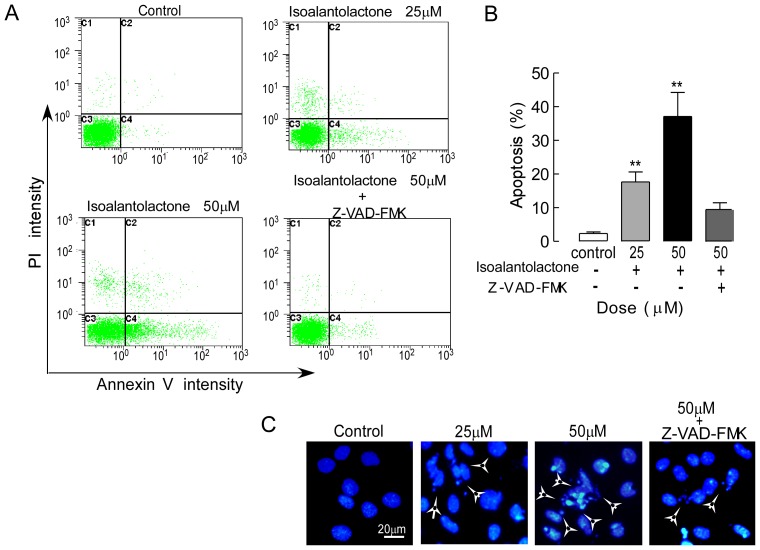
Isoalantolactone-induced apoptosis in UM-SCC-10A cells. (A) Apoptosis was evaluated using an annexin V-FITC apoptosis detection kit and flow cytometry. The X- and Y-axes represent annexin V-FITC staining and PI, respectively. The representative pictures are from UM-SCC-10A cells incubated with different concentrations of isoalantolactone (25 and 50 µM) or caspase inhibitor (Z-VAD-FMK 50 µM). (B) Isoalantolactone induced apoptosis in the UM-SCC-10A cells in a dose-dependent manner. Z-VAD-FMK markedly reduced apoptosis in UM-SCC-10A cells treated with high-dose isoalantolactone. The data are expressed as the means ± SEM of three independent experiments with the similar results. *P<0.05 and **P<0.01 compared to the control. (C) The morphological nuclear changes in UM-SCC-10A cells treated with isoalantolactone at different concentrations. The cells were stained with Hoechst33258 for 30 min in the dark to examine the cleaved nuclei, which is a sign of apoptosis.

First, we determined whether isoalantolactone-induced cell death was caused by apoptosis or necrosis. Phosphatidylserine externalization, a hallmark of early phase apoptosis, was analyzed using annexin V/PI double-labeling and flow cytometry. The results indicated that the percentage of annexin V-positive cells increased after treatment with isoalantolactone at different concentrations compared to control cells (Figure 2A). However, no significant early or late apoptosis was observed in the UM-SCC-10A cells pretreated with Z-VAD-FMK, an extensive caspase inhibitor. These results suggest that isoalantolactone induced UM-SCC-10A cell apoptosis possibly via the caspase pathway.

To further verify this isoalantolactone-induced apoptotic cell death in UM-SCC-10A cells, we analyzed morphological nuclear changes using Hoechst33258 staining. DNA fragmentation and loss of plasma membrane asymmetry are the most typical characteristics of apoptotic cell death. Figure 2B shows increased nuclear shrinkage, condensation and DNA fragmentation in isoalantolactone-treated cells compared to control cells. Cells pretreated with Z-VAD-FMK had no significant nuclear change following isoalantolactone treatment at different concentrations. Our data indicate that caspase was involved in isoalantolactone-induced UM-SCC-10A cell apoptosis.

### Isoalantolactone Induces Cell Cycle Arrest-independent Apoptosis

**Figure 3:**
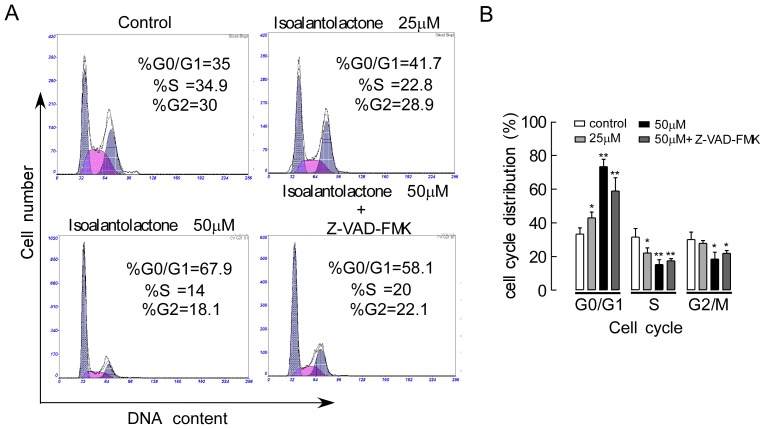
The effect of isoalantolactone on the cell cycle in UM-SCC-10A cells. (A) The DNA content in each cell cycle phase in UM-SCC-10A cells was analyzed by flow cytometry. The representative histograms are from UM-SCC-10A cells incubated with different concentration of isoalantolactone (25 and 50 µM) or caspase inhibitor (Z-VAD-FMK50 µM). (B) Isoalantolactone treatment induced a dose-dependent increase in the proportion of cells in the G1 phase and a decrease in cells in the S and G2 phases compared to the control. Z-VAD-FMK treatment did not prevent cell cycle arrest following high-dose isoalantolactone treatment. The results are represented as the mean ± SEM for three independent experiments with similar results. *P<0.05 and **P<0.01 compared to the control.

Cell cycle arrest is one of the major causes of cell death. To explore whether isoalantolactone-induced apoptosis was associated with cell cycle arrest, we first examined cell cycle distribution in UM-SCC-10A cells using flow cytometry to analyze the DNA content in each cell cycle phase. In a dose-dependent manner, there was a progressive increase in the percentage of cells in the G1 phase after the UM-SCC-10A cells were exposed to isoalantolactone at different concentrations, whereas there was a significant decrease in the number of cells in the S and G2 phases (Figure 3). Furthermore, we used a general caspase inhibitor Z-VAD-FMK for the apoptosis and cell cycle analyses in UM-SCC-10A cells. Flow cytometric analysis showed Z-VAD-FMK did not prevent cell cycle arrest in a specific phase, although isoalantolactone induced UM-SCC-10A cell apoptosis. These data indicate that isoalantolactone induced UM-SCC-10A cell death through cell cycle arrest in G1-phase and by the induction of apoptosis.

### Isoalantolactone Induces the Expression of Cell Cycle Regulators

**Figure 4:**
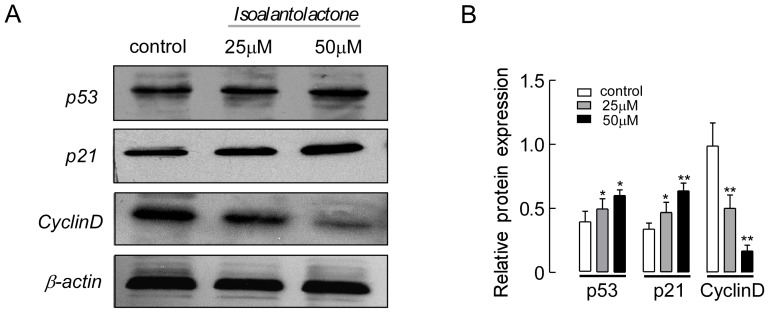
The effect of isoalantolactone on the expression of cell cycle regulators in UM-SCC-10A cells. (A) Representative pictures for p53, p21 and cyclin D protein expression by western blot analysis. β-actin was used as a control. (B) Isoalantolactone up-regulated p53 and p21 expression, while down-regulating cyclin D expression in a dose-dependent manner. The results are represented as the means ± SEM from three independent experiments with similar results. *P<0.05 and **P<0.01 compared to the control.

Based on the results described above, isoalantolactone induced UM-SCC-10A cell cycle arrest. The tumor suppressor protein, p53, regulates the cell cycle, and its target gene, p21, directly inhibits cyclin D. This pathway results in cell cycle arrest in the G1 phase. To investigate the mechanism of isoalantolactone-induced cell cycle arrest in UM-SCC-10A cells, we analyzed p53, p21 and cyclin D expression by western blotting. As shown in Figure 4, the expression levels of p53 and p21 were markedly increased, whereas cyclin D expression was significantly decreased in a dose-dependent manner. These results reveal that isoalantolactone induced cell death in the UM-SCC-10A cells through p53 activation, leading to cell cycle arrest in the G1 phase.

### Isoalantolactone Induces Apoptosis in the Mitochondria

**Figure 5:**
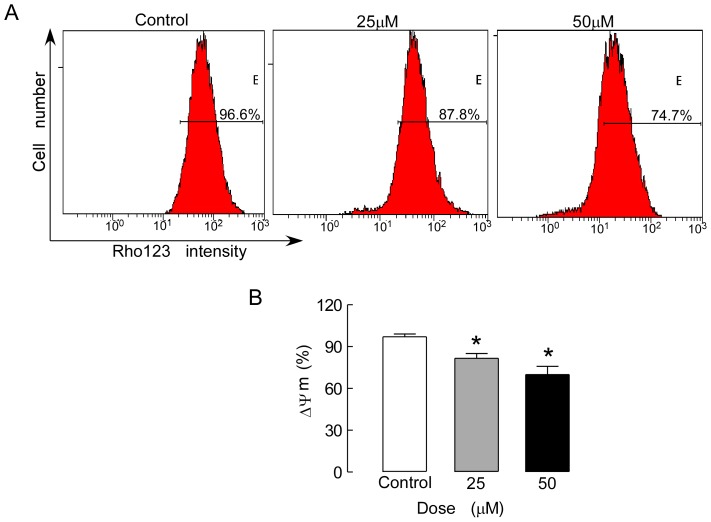
The effect of isoalantolactone on the MMP in UM-SCC-10A cells. (A) The MMP of UM-SCC-10A cells treated with isoalantolactone at different concentrations was analyzed by flow cytometry. (B) The loss of the MMP in UM-SCC-10A cells following isoalantolactone treatment in a dose-dependent manner. The data are expressed as the means ± SEM for three independent experiments with similar results. *P<0.05 compared to the control.

To investigate whether isoalantolactone-induced cell apoptosis was associated with mitochondrial dysfunction, we analyzed MMP changes in the UM-SCC-10A cells by staining with Rho123, a mitochondria-sensitive dye, and analyzing the cells by flow cytometry. Another characteristic feature of apoptosis is depolarization of the mitochondrial membrane potential. Our results showed that the MMP of the UM-SCC-10A cells decreased significantly after treatment with isoalantolactone in a dose-dependent manner, suggesting that isoalantolactone induced cell apoptosis through the intrinsic pathway (Figure 5).

### Isoalantolactone Induces Cell Apoptosis through a Mitochondrial Pathway

**Figure 6:**
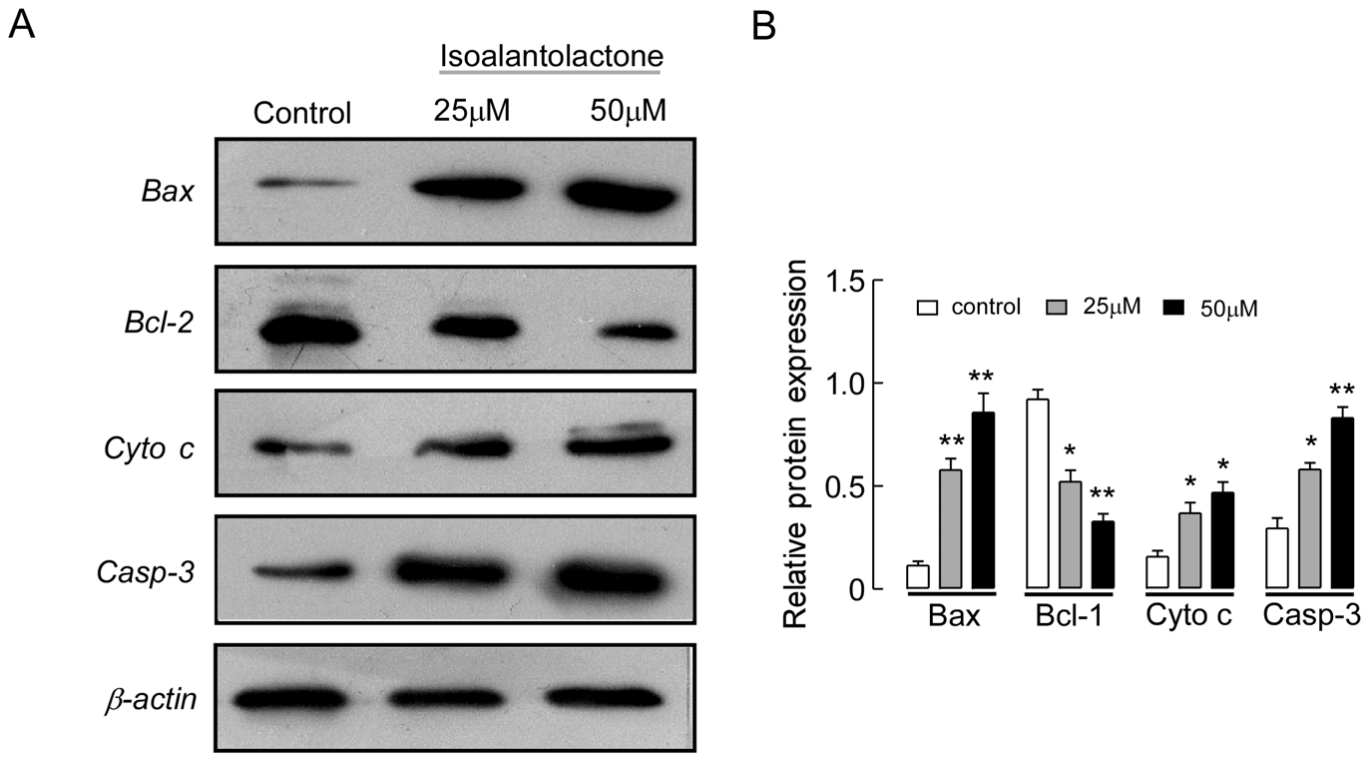
The effect of isoalantolactone on the expression of caspase-dependent mitochondrial apoptosis pathway proteins in UM-SCC-10A cells. (A) Representative images of cytochrome c, Bax, Bcl-2 and caspase 3 protein expression detected by western blot. β-actin was used as a control. (B) The data are represented as the means ± SEM from three independent experiments with similar results. *P<0.05 and **P<0.01 compared to the control.

Mitochondrial damage facilitates cytochrome c release from mitochondria into the cytoplasm and activates apoptotic factors (Bcl-2 family proteins), which leads to activation of the caspase cascade (apoptotic markers) and mitochondrial apoptosis. To test whether isoalantolactone induces apoptosis through this mechanism in UM-SCC-10A cells, we examined the expression of cytochrome c, the pro-apoptotic protein Bax, the anti-apoptotic protein Bcl-2, and caspase 3 by western blot analysis. In a dose-dependent manner, isoalantolactone significantly increased cytosolic cytochrome c, Bax and caspase 3 expressions with a concomitant decrease in Bcl-2 expression compared to the control group (Figure 6). These results suggest that the mitochondria and Bcl-2 family members are involved in isoalantolactone-mediated cell apoptosis in UM-SCC-10A cells.

## Discussion

Previous studies have indicated that the root extract of *Inula helenium* L. has ideal natural compounds for targeted treatment of many cancer cell lines [11], [12]. Although some studies have demonstrated anticancer activity for the sesquiterpene lactones, which are isolated from the roots of *Inula helenium* L., in various human tumor cell lines, their anti-HNSCC potential is still unknown [6]. Isoalantolactone is one of the major sesquiterpene lactone compounds. In the present study, we investigated the anti-tumor effects and mechanism of action of isoalantolactone in UM-SCC-10A cells. Our data demonstrated that isoalantolactone markedly inhibited UM-SCC-10A cell growth in a time- and dose-dependent manner at low doses. The ideal cancer chemotherapeutic drug would have minimal adverse effects on normal cells [13]. Interestingly, we found that the growth-inhibiting effect of isoalantolactone is tumor cell selective, as normal mouse splenocytes did not display a significant toxic effect *in vitro*.

Cell cycle regulation and apoptosis are two major regulatory mechanisms for cell growth. When specific checkpoints during the cell cycle are arrested, apoptotic cell death occurs [14]–[19]. Moreover, many chemotherapeutic agents cause cell cycle arrest through microtubule damage and have been proven to be clinically effective for treating cancer [20], [21]. Thus, we evaluated whether isoalantolactone affects these two mechanisms using a series of assays. In our study, isoalantolactone induced cell cycle arrest as shown by PI staining and flow cytometry. Flow cytometric studies showed that the growth inhibition induced by isoalantolactone in a dose-dependent manner occurs through the arrest of the UM-SCC-10A cells in the G1 phase.

Additionally, p53, the most extensively studied tumor suppressor, mediates a variety of anti-proliferative processes through cell cycle checkpoints, DNA repair and apoptosis [22]. Some previous reports have found that p21, the target of p53, is one of the major cyclin-dependent kinase inhibitors (CDKIs), which directly inhibit the activity of specific CDKs, thereby leading to cell cycle arrest through a p53-dependent pathway. Upregulation of p21 expression may inhibit cyclin/CDK complexes, thus leading to cell cycle arrest [23]–[27]. To gain further insight into the molecular mechanisms underlying isoalantolactone-induced G1 arrest in UM-SCC-10A cells, we examined p53, p21 and cyclin D expression. Western blot analysis revealed that isoalantolactone significantly increased p53 and p21 expression in UM-SCC-10A cells, which was consistent with the suppressed expression of the cell cycle-related protein cyclin D.

To determine whether isoalantolactone-induced inhibition of UM-SCC-10A cell growth is dependent on apoptosis, we performed flow cytometric analysis of apoptosis after treating with isoalantolactone. Our data showed that isoalantolactone induced UM-SCC-10A cell apoptosis in a dose-dependent manner. Furthermore, after pre-incubation with Z-VAD-FMK, an extensive caspase inhibitor, apoptosis was greatly attenuated indicating that apoptosis occurs via a caspase-dependent pathway. Moreover, we also found that inhibition of caspase activation did not prevent mitotic arrest, suggesting that isoalantolactone inhibited UM-SCC-10A cell growth via either cell cycle arrest or apoptosis induction.

Apoptosis occurs through two main pathways: the death receptor pathway and the intrinsic or mitochondrial pathway [28]. The mitochondrial and Bcl-2 family proteins are key factors in the apoptotic signaling pathway. Previous studies have shown that the Bcl-2 family proteins are usually involved in the mitochondrial apoptotic signal pathway [29]. The Bcl-2 family includes both anti-apoptotic (Bcl-2) and pro-apoptotic (Bax) proteins. The ratio of Bcl-2 and Bax regulates cytochrome c to activate the caspase cascade through the mitochondrial transition pore (PTP), leading to activation of caspase 3. Caspase 3 has been identified as a key mediator of apoptosis in mammalian cells [30]–[31].

Involvement of the mitochondrial pathway in Bcl-2-mediated apoptosis was first confirmed by observing changes in MMP and Bcl-2 and Bax protein expression after isoalantolactone treatment. Mitochondrial dysfunction often involves MMP loss and cytochrome c release from mitochondria into the cytosol [32], [33]. We found that isoalantolactone significantly decreased the MMP, causing cytochrome release from the mitochondria into the cytoplasm. This result suggests that isoalantolactone-induced cell apoptosis correlates with mitochondrial dysfunction. Moreover, western blot analysis indicated that isoalantolactone significantly decreased Bcl-2 expression and increased Bax expression, leading to an increased Bax/Bcl-2 ratio in UM-SCC-10A cells. Therefore, we concluded that isoalantolactone can promote opening of the mitochondrial PTP by increasing the Bax/Bcl-2 ratio. Next, we assessed the effect of isoalantolactone on caspase 3 activation in UM-SCC-10A cells. Our results revealed that isoalantolactone markedly increased caspase 3 expression in a dose-dependent manner. Furthermore, pretreatment with Z-VAD-FMK, an extensive caspase inhibitor, resulted in a significant decrease in apoptosis, indicating that caspase 3 is involved isoalantolactone-induced apoptosis. These results suggest that isoalantolactone induces caspase-dependent apoptosis via the mitochondrial pathway.

In conclusion, our study suggests that isoalantolactone exerted an inhibitory effect on UM-SCC-10A cells by arresting the cell cycle in the G1 phase and inducing apoptosis. Furthermore, G1 phase arrest was found to be associated with the up-regulation of p53 and p21 and the down-regulation of cyclin D. In addition, the induction of apoptosis was associated with disruption of MMP, release of cytochrome c and modulation of Bcl-2 family proteins. Thus, isoalantolactone could be a promising chemotherapeutic drug candidate for treating head and neck squamous cell carcinoma.
